# Attention-enhanced deep learning model for MQTT intrusion detection in internet of medical things

**DOI:** 10.3389/frai.2026.1934767

**Published:** 2026-09-16

**Authors:** Siddhartha Singhal, Kakelli Anil Kumar

**Affiliations:** School of Computer Science and Engineering, Vellore Institute of Technology, Vellore, Tamil Nadu, India

**Keywords:** attention mechanism, CICIoMT2024 dataset, deep learning, Internet of Medical Things (IoMT), intrusion detection system, MQTT

## Abstract

**Introduction:**

The increasing utilization of the Message Queuing Telemetry Transport (MQTT) protocol in IoMT has created a high demand for efficient intrusion detection systems (IDSs).

**Methods:**

This study proposes a protocol-specific intrusion detection system (IDS) that employs a hybrid deep learning architecture consisting of a one-dimensional convolutional neural network along with a multi-head self-attention mechanism (CNN-MHSA). The architecture employs a convolutional feature-extraction module to derive local traffic features and a multi-head self-attention mechanism to capture long-range feature correlations. The proposed approach is evaluated on the CICIoMT2024 dataset, focusing mainly on the MQTT attack scenarios that are framed as a binary classification problem, in which five distinct MQTT attack variants (DoS-Connect Flood, DDoS-Connect Flood, DoS-Publish Flood, DDoS-Publish Flood, and Malformed Data) were consolidated into a single attack class against benign traffic.

**Results:**

The test results show an accuracy of 99.40%, an F1-score of 99.52%, and an AUC of 0.9998, respectively, for the proposed method.

**Discussion:**

The results indicate that the proposed CNN–MHSA framework offers a robust benchmark for secure healthcare communication and can successfully identify MQTT threats in IoMT networks.

## 1 Introduction

Internet of Medical Things (IoMT) technology is continuously evolving and reshaping how patient follow-up, diagnosis, and decision-making are executed by connecting devices and networks (Praveenkumar et al., 2026). The IoMT poses a significant challenge to cybersecurity, however, due to the growing interconnection of various medical devices and limited resources in decentralized environments where valuable healthcare information is shared. Compromised devices or malicious network activity may result in unauthorized information access, tampering vital medical information, and compromise of critical services, thus requiring the incorporation of robust intrusion detection framework to ensure the safe usage of IoMT technologies (Wani et al., 2024; Sharma et al., 2026).

The MQTT protocol, known for its simplicity and efficiency in implementing a publish-subscribe paradigm (Alshammari, 2023) is widely used in various IoMT applications. There are serious bandwidth and power constraints for medical devices, which make MQTT a highly favored choice for the IoMT system. The advantages of the MQTT protocol, however, as well as the centralized communication paradigm it provides, constitute severe cybersecurity threats in IoMT scenarios given the potential for DoS/DDoS attacks, spoofing, unauthorized publishing, and invalid packet injection. DoS and DDoS attacks can affect the integrity of IoMT networks as MQTT brokers play a crucial role in these networks (Hintaw et al., 2023).

In response to these challenges, many machine learning-based, hybrid learning-based, and deep learning-based intrusion detection methods have been proposed. While conventional machine learning methods can be competitive in a controlled environment, they tend to involve extensive feature engineering and do not have a strong ability to model complex nonlinear relationships under changing network traffic conditions. More recently, deep learning architectures, including convolutional neural networks (CNNs), recurrent neural networks (RNNs), attention-based models, transformer networks, and graph neural networks, have demonstrated substantial improvements in automated feature learning and intrusion detection performance. Nevertheless, many existing architectures primarily focus on temporal sequence modeling, graph representation learning, or general-purpose intrusion detection and are commonly evaluated using benchmark datasets that do not fully represent contemporary IoMT communication environments (Ullah et al., 2024). Furthermore, comparatively limited attention has been given to integrating lightweight convolutional feature extraction with multi-head self-attention (MHSA) for protocol-specific MQTT intrusion detection using the recently introduced CICIoMT2024 dataset, while simultaneously evaluating computational efficiency, architectural effectiveness, and cross-dataset generalization. The principal findings of the research are outlined below:

- A protocol-specific intrusion detection framework is developed by isolating MQTT-specific attack traffic from the heterogeneous CICIoMT2024 dataset, enabling a focused binary classification framework tailored to IoMT communication behavior.
- A lightweight 1D CNN-MHSA architecture is proposed to jointly capture hierarchical local traffic patterns through one-dimensional convolution and long-range feature dependencies through multi-head self-attention, providing an effective representation for protocol-specific MQTT intrusion detection.
- A comprehensive preprocessing methodology is developed to identify and consolidate all MQTT attack variants, resulting in a large-scale binary classification dataset containing 5,56,992 network flow instances characterized by 45 statistical features.
- Extensive experimental evaluation, including ablation studies, computational efficiency analysis, and cross-dataset validation, demonstrates the effectiveness and generalization ability of the proposed methodology, achieving an AUC of 0.9998 on the CICIoMT2024 MQTT dataset.

The remainder of this work is structured as follows. Section 2 reviews recent research on MQTT-based intrusion detection. Section 3 presents the dataset preprocessing methodology and the proposed CNN–MHSA framework. Section 4 reports the experimental setup and performance evaluation. The results are discussed in Section 5, and the work is concluded, and future study goals are outlined in Section 6.

## 2 Related work

Although cyberattacks targeting lightweight IoT protocols are becoming more frequent, there has been an increase in interest in intrusion detection for IoMT networks, especially those using the MQTT communication protocol. Recent studies have explored a wide range of intrusion detection approaches, including conventional machine learning, hybrid ensemble learning, deep learning, attention-based architectures, transformer networks, and graph neural networks. Table 1 summarizes representative machine learning and deep learning methods related to MQTT- and IoMT-based intrusion detection.

**Table 1:** An overview of representative machine learning and deep learning approaches for MQTT and IoMT intrusion detection.

Author	Dataset	Model	Accuracy (%)	Key contribution
Siddharthan et al. (2022)	SENMQTT-SET	Ensemble ML models	100	Evaluation of various machine learning models utilizing the designed attributes
Alasmari and Alhogail (2024)	MQTT-IoT-IDS2020	Multiple ML algorithms	100	Automated feature engineering improves MQTT IDS performance
Alatram et al. (2023)	DoS/DDoS-MQTT-IoT	Multiple ML models	100	Introduced an MQTT attack dataset that encompassed several attack scenarios.
Zeghida et al. (2025a)	MQTT-IoT-IDS2020	RF, SVM, CNN-LSTM	99.99	Explainable Machine Learning Intrusion Detection Systems Utilizing SHAP and LIME
Umer et al. (2025)	UNSW-NB15	ML with Multi-Head Attention	93	Attention-enhanced ML model for complex attack patterns
Solanki and Gupta (2025)	MQTT-IoT-IDS2020	Stacked Ensemble (SVM, MLP, NB)	99.80	Meta-learning ensemble improves MQTT attack detection
Dandapat and Mondal (2024)	MQTT-IoT-IDS2020	GA with CNN	99.51	Genetic feature selection enhances CNN performance
Pawar et al. (2024)	Real IoT testbed	Deep Neural Network	99.98	QoS-aware MQTT intrusion detection using real hardware
Hernandez-Jaimes et al. (2025)	CICIoMT2024	DNN and OC-SVM	91.81	One-class SVM combined with DNN for anomaly detection in IoMT networks
Shaikh et al. (2024a)	ICU and WUSTL-EHMS 2020	FOID	98.53	Feature optimization using SMOTE and XGBoost-RFE for IoMT intrusion detection
Shaikh et al. (2025a)	CICIoMT2024	HCLR-IDS	99.58	Hybrid CNN-LSTM with deep reinforcement learning for binary and multiclass IoMT intrusion detection
Alshehri et al. (2024)	IoTID20, Edge-IIoTset	SA-DCNN	96.89	Self-attention CNN improves feature importance learning
Ullah et al. (2023a)	Edge-IIoTset	MAGRU-IDS	99.99	Multi-head attention with GRU handles class imbalance
Hu et al. (2024)	CICIDS2017	SAGB-BiGRU	98.40	Combines temporal and attention-based feature extraction
Shaikh et al. (2024b)	WUSTL-EHMS-2020	RCLNet	99.78	CNN-LSTM with self-adaptive attention for spatial–temporal feature learning
Alblehai (2025)	ToN-IoT	Self-Attention IDS	99.44	Attention-based deep learning for high-dimensional IoT data
de Araujo-Filho et al. (2023)	CICDDoS2019	GAN, TCN, and Self-Attention	97.07	Unsupervised IDS requiring only benign training data
Shaikh et al. (2025b)	WUSTL-EHMS-2020, ECU-IoHT, and X-IIoTID	Memory Feedback Transformer	99.98	Transformer enhanced with recurrent memory for contextual representation learning
Ullah et al. (2023b)	MQTT-IoT-IDS2020	Transformer IDS	99.99	Transformer-based IDS for MQTT traffic
Akuthota and Bhargava (2025)	NSL-KDD, UNSW-NB15	Transformer IDS	99.97	Efficient transformer architecture for intrusion detection
Reka et al. (2024)	MANET simulation	MSA-GCNN	97.12	Graph attention model for multi-attack detection
Shaikh et al. (2023)	UAV-assisted IoMT	Stackelberg Game	N/R	Game-theoretic adversarial defense for securing IoMT healthcare networks

The reported accuracy values correspond to the highest classification accuracy reported in the respective original publications.

The SENMQTT-SET framework, developed by Siddharthan et al. (2022), evaluates the effectiveness of several machine learning techniques on the SENMQTT-SET dataset, including LR, KNN, SVM, NB, DT, RF, and GB. This framework shows that employing classifiers with feature engineering techniques can yield close to maximum detection performance in detecting MQTT-based flooding attacks. Alasmari and Alhogail (2024) proposed various machine learning algorithms on the MQTT-IoT-IDS2020 dataset, which uses automated feature engineering techniques to boost detection accuracy in the IoT-based smart home. Alatram et al. (2023) introduced a significant contribution to MQTT-based intrusion detection systems by proposing the DoS/DDoS-MQTT-IoT dataset, which can be used to evaluate intrusion detection algorithms for MQTT-based denial-of-service attacks. The analysis results of this study show that conventional models, such as DT, SVM, and GB, can be used to identify the MQTT-based DOS attack. To enhance the efficacy of identifying intrusion in MQTT-based IoT applications, Zeghida et al. (2025a) presented the XMID-MQTT system that integrates several machine learning methods, like RF, SVM, and CNN-LSTM, with explainability methods like SHAP and LIME. Umer et al. (2025) introduced an innovative machine learning approach that integrates a feature selection method with a multi-head attention system to enhance the detection of complicated attack patterns on the UNSW-NB15 dataset.

Numerous studies have previously examined hybrid and ensemble learning models to provide the requisite resilience in detection, employing a combination of several classifiers and feature optimization strategies. Solanki and Gupta (2025) presented a stacked ensemble-based intrusion detection system, with efficiency assessed using the MQTT-IoT-IDS2020 dataset. A meta-learning classifier was applied to integrate various base classifiers (SVM, MLP, Naïve Bayes, etc.) using logistic regression. This meta-learning architecture was shown to be very effective in the proposed stacked-ensemble-based intrusion detection system, achieving a notable enhancement in intrusion detection accuracy. Dandapat and Mondal (2024) suggested a hybrid intrusion detection that combines a genetic algorithm along with a convolutional neural network. The fusion IDS achieved better performance in detecting attacks on the MQTT-IoT-IDS2020 dataset.

Pawar et al. (2024) introduced an intrusion detection using a deep neural network that employs a genuine IoT hardware testbed. The proposed architecture was founded on multiple quality-of-service attributes, including packet delay, packet loss, and authentication, to identify intrusions in MQTT communication. Hernandez-Jaimes et al. (2025) presented a hybrid architecture that incorporates a deep neural network and a one-class SVM for identifying anomalies. The proposed architecture has been evaluated and shown to have excellent performance in detecting abnormalities in the IoT network using CICIoMT2024 and MQTT-IoT-IDS2020 datasets. Shaikh et al. (2024a) proposed the Feature-Optimized Intrusion Detection (FOID) framework, which integrates SMOTE-based class balancing with XGBoost-based recursive feature elimination to improve machine learning-based intrusion detection in IoMT healthcare networks. More recently, Shaikh et al. (2025a) introduced HCLR-IDS, a hybrid framework combining CNN, LSTM, and deep reinforcement learning for intrusion detection on the CICIoMT2024 dataset, demonstrating the growing adoption of hybrid deep learning architectures for healthcare-oriented IoMT security.

Building upon these hybrid deep learning approaches, recent research has increasingly incorporated attention mechanisms to improve feature representation and capture long-range dependencies in network traffic. Using the IoTID20 and Edge-IIoTset datasets, Alshehri et al. (2024) tested a DCNN that uses a self-attention mechanism (SA-DCNN). The proposed model relies on attention mechanisms and a deep CNN, which enables improved learning of feature importance in high-dimensional network traffic data, thereby enhancing its performance in accurately detecting anomalies and intrusions in the datasets used for evaluation. Ullah et al. (2023a) presented a novel intrusion detection system, termed MAGRU-IDS, which utilizes multi-head attention and gated recurrent units to enhance the identification of imbalanced classes in IoT network traffic data. Hu et al. (2024) introduced a model termed SAGB-BiGRU, which combines self-attention and bidirectional recurrent neural networks to better capture temporal relationships and global feature interactions. Shaikh et al. (2024b) further proposed RCLNet, which integrates Random Forest-based feature selection, a CNN-LSTM backbone, and a self-adaptive attention mechanism to jointly learn spatial and temporal characteristics of IoMT traffic.

Alblehai (2025) conducted further research in the area of attention-driven deep learning with the integration of metaheuristic optimization algorithms for efficient intrusion detection in large-scale IoT environments. de Araujo-Filho et al. (2023) presented a similar method, proposing an unsupervised intrusion detection method based on GAN, temporal convolutional networks, and self-attention mechanisms, which only employs benign traffic samples for training. Shaikh et al. (2025b) introduced a memory-feedback transformer architecture that enhances conventional transformer models through recurrent memory mechanisms for improved contextual feature representation in IoMT intrusion detection. These studies demonstrate that attention mechanisms substantially improve feature representation and long-range dependency learning, motivating the development of increasingly sophisticated attention-driven intrusion detection architectures. Ullah et al. (2023b) presented a transformer-based methodology utilizing the MQTT-IoT-IDS2020 dataset, attaining elevated accuracy, precision, recall, and F1 scores, thereby demonstrating its efficacy in the IoT domain. Similarly, Akuthota and Bhargava (2025) introduced an intrusion identification system based on a transformer, which is trained on the NSL-KDD and UNSW-NB15 datasets and successfully generalized over multiple intrusion detection benchmarks. To recognize different kinds of attacks in mobile ad-hoc networks, Reka et al. (2024) introduced a multi-head self-attention (MSA) gated graph convolution network (GCNN). Inspired by these detection methods, Shaikh et al. (2023) introduced a Stackelberg game-theoretic framework for securing UAV-assisted IoMT healthcare networks, which models the interactions between the attacker and defender to enhance the network’s resilience against adversarial attacks.

### 2.1 Summary of research gap

Although considerable progress has been achieved in MQTT intrusion detection through conventional machine learning, hybrid ensemble learning, deep neural networks, attention-based architectures, transformer models, and graph neural networks, several challenges remain. Conventional machine learning and ensemble methods heavily rely on feature engineering and frequently fail to capture complex nonlinear relationships in evolving IoMT traffic, whereas many hybrid deep learning models increase architectural complexity or are developed for general IoT environments rather than protocol-specific MQTT communication.

Recent attention-based approaches, including SA-DCNN, MAGRU-IDS, SAGB-BiGRU, TNN-IDS, transformer-based IDS, memory-feedback transformers, RCLNet, HCLR-IDS, and MSA-GCNN, have demonstrated the effectiveness of attention mechanisms, recurrent architectures, reinforcement learning, and graph-based representations for improving intrusion detection performance. However, these models primarily focus on temporal sequence modeling, class imbalance mitigation, graph-based representation learning, reinforcement learning, or general-purpose IoMT intrusion detection and are mostly evaluated using datasets such as MQTT-IoT-IDS2020, Edge-IIoTset, NSL-KDD, UNSW-NB15, MANET simulations, or heterogeneous IoMT traffic. Consequently, their applicability to protocol-specific MQTT intrusion detection in contemporary IoMT environments represented by the CICIoMT2024 dataset remains insufficiently explored. Furthermore, although recent transformer-, recurrent attention-, reinforcement learning-, and graph-based architectures have demonstrated strong capability for learning complex feature relationships, limited attention has been given to integrating lightweight one-dimensional convolution for hierarchical local feature extraction with multi-head self-attention within a lightweight feed-forward architecture specifically designed for protocol-isolated MQTT intrusion detection.

To overcome these gaps, this work proposes: (1) a protocol-specific MQTT intrusion detection framework; (2) a lightweight 1D CNN-MHSA architecture that jointly captures local feature patterns and global feature dependencies; and (3) comprehensive evaluation on the CICIoMT2024 dataset, supported by ablation analysis, computational efficiency assessment, and cross-dataset validation.

## 3 Proposed methodology

### 3.1 Dataset description

The CICIoMT2024 dataset is an extensive compilation of data designed for the assessment and evaluation of intrusion detection systems within Internet of Medical Things (IoMT) contexts, achieved by simulating network traffic across various IoT and IoMT applications and scenarios (Dadkhah et al., 2024). The dataset effectively simulates real-world network conditions, as it contains data from 40 IoT devices, communicating via different channels, such as Wi-Fi, Bluetooth, and MQTT. It contains 18 types of attacks, such as DoS/DDoS, spoofing, espionage, and MQTT-specific attacks, and over 8.7 million network flow records classified by statistical traffic features. Figure 1 shows notable class imbalance in the dataset across multiple attack types, with large-scale attacks, such as DDoS on TCP/IP networks, making up a large proportion of the dataset (millions of instances), whereas attacks involving MQTT constitute a small proportion. This high degree of heterogeneity underscores the challenge of acquiring general intrusion patterns and motivates the protocol-specific filtering approach adopted in this study, allowing the focus to be placed on MQTT threats.

**Figure 1:**
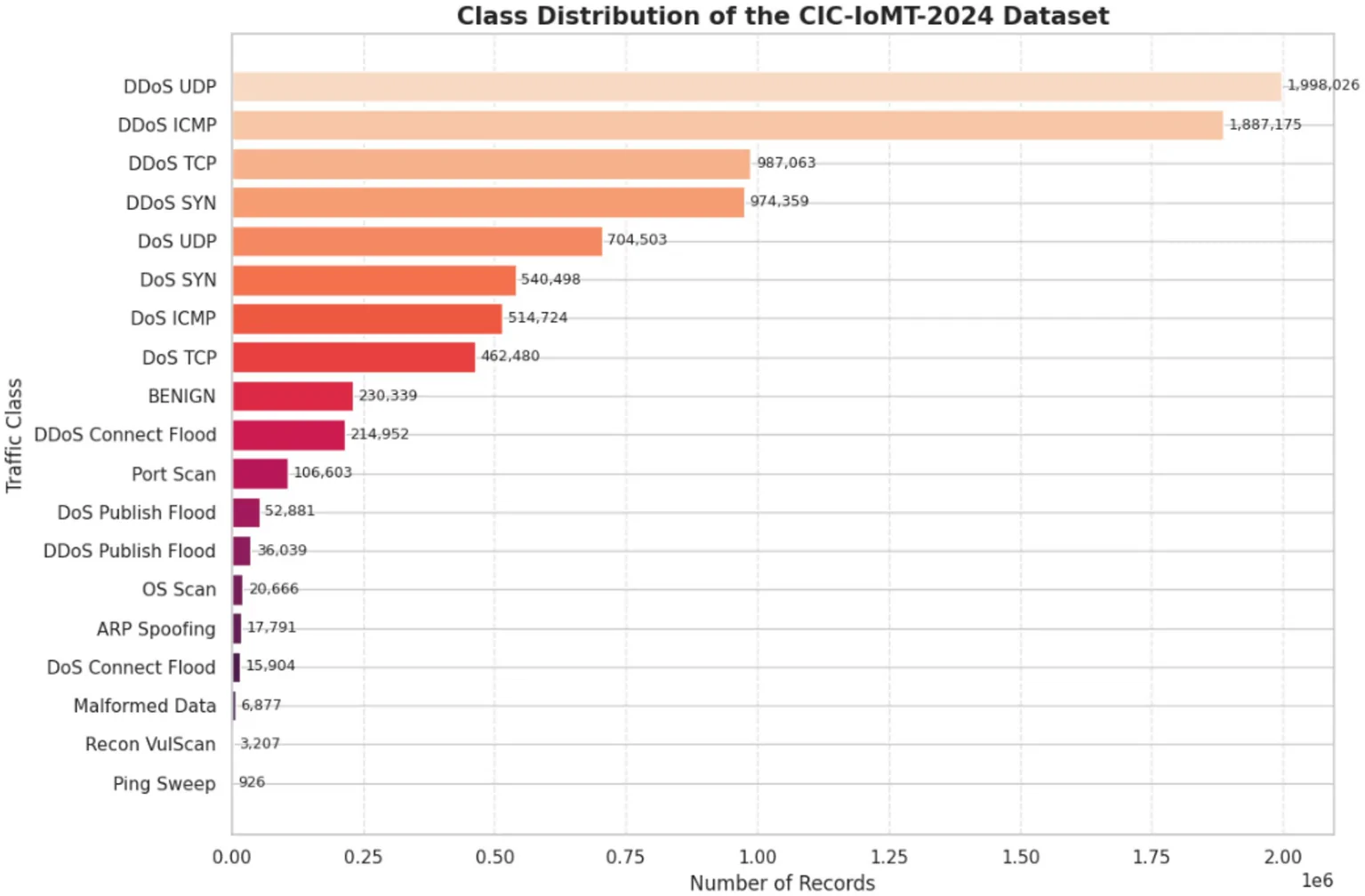
Class-wise distribution of traffic instances in the CICIoMT2024 dataset.

### 3.2 Data preprocessing and dataset preparation

The central processing pipeline was utilized on the CICIoMT2024 dataset to produce protocol-specific intrusion detection data, as illustrated in Algorithm 1. The primary objective is to extract MQTT-specific attack traffic and convert the multi-class classification issue to a binary classification task.

#### 3.2.1 Attack filtering and dataset construction

The CICIoMT2024 dataset includes numerous types of attacks on different protocols and protocol layers. In particular, this study focuses on MQTT-related attacks because Message Queuing Telemetry Transport is extensively utilized in IoMT systems and is susceptible to flooding and malformed-packet attacks. A protocol-based filtering strategy was used to separate MQTT-related attack traffic and address vulnerabilities related to flooding and malformed-packet attacks in IoMT systems. The MQTT-related portion of CICIoMT2024 comprises five attack subsets, denoted ${D_{i}}^{*}=Z_{i}\gamma +\delta_{i}$ through $A_{5}$ : MQTT-DoS-Connect Flood ( $A_{1}$ ), MQTT-DDoS-Connect Flood ( $A_{2}$ ), MQTT-DoS-Publish Flood ( $A_{3}$ ), MQTT-DDoS-Publish Flood ( $A_{4}$ ), and MQTT-Malformed Data ( $A_{5}$ ). To establish a binary classification problem, all MQTT attack variations were consolidated into a single attack class, denoted as $C_{\text{attack}}$ , as specified in Equation 1:

$$C_{\text{attack}}=\cup_{i=1}^{5}A_{i}$$ (1)

where $A_{i}\in$ {DoS-Connect, DDoS-Connect, DoS-Publish, DDoS-Publish, Malformed}.

Benign traffic samples were retained as a separate class without modification. This combination of samples enabled the model to acquire generalized intrusion patterns for the MQTT protocol. Simultaneously, this avoids fragmentation of data into different classes of attacks, which could lead to a less accurate analysis of the vulnerabilities of the MQTT protocol. Filtered protocol-specific samples effectively eliminated the interference of non-MQTT attacks, ensuring the analysis focused solely on vulnerabilities inherent to the MQTT protocol.

#### 3.2.2 Dataset statistics

The dataset was then arranged according to the binary classification paradigm of benign and MQTT attacks. The dataset was divided into training and testing sets. The testing dataset included 1,01,322 network flows, of which 37,607 samples represented benign network flows, and 63,715 samples represented network flows related to attacks. The training data consisted of 4,55,670 network flows, 1,92,732 of which were benign samples and 2,62,938 were samples of MQTT attacks, as shown in Table 2.

**Table 2:** Statistical summary of the MQTT binary dataset.

Dataset partition	Benign	MQTT attack	Total	Number of features
Training set	192,732	262,938	455,670	45
Test set	37,607	63,715	101,322	45
Total	230,339	326,653	556,992	45

Formally, the dataset is specified as Equation 2:

$$D={\left\{(x_{i},y_{i})\right\}}_{i=1}^{N}$$ (2)

Here $x_{i}\in {\mathbb{R}}^{45}$ indicates a feature vector, and $y_{i}\in \{0,1\}$ denotes the class label (0 for benign, 1 for attack). The total dataset size was N=556,992 .

#### 3.2.3 Feature representation

Each network flow is represented by 45 statistical features related to the IoMT transmission characteristics. These statistical features are related to the length of network flows, packet characteristics, inter-arrival times of packets, and network flow rates. Thus, each network flow instance is represented as a 45-dimensional feature vector, defined in Equation 3:

$$x=[f_{1},f_{2},\ldots ,f_{45}]$$ (3)

where $f_{j}$ denoted the $j^{\mathrm{th}}$ feature of the network flow.

Min-max normalization was employed on all 45 numerical features to maintain numerical stability during model training, scaling each feature to the range [0, 1], as delineated in Equation 4:

$${\hat{x}}_{j}=\frac{f_{j}-f_{j,\min}}{f_{j,\max}-f_{j,\min}+\varepsilon }$$ (4)

where $f_{j,\min}$ and $f_{j,\max}$ denote the minimum and maximum values of the feature $f_{j}$ observed in the training set, and $\varepsilon =10^{-8}$ is a minor constant introduced for statistical stability. Normalization variables were extracted only from the training data and subsequently employed in the validation and test sets to prevent data leakage. Prior to normalization, all numerical features were examined for missing and non-finite values. As summarized in Table 3, no missing values were identified in the MQTT-filtered subset. A total of 742 positive infinite values (0.0030% of the 25,064,640 feature values) were detected across 611 network flows, originating exclusively from the Rate, Srate, and Drate features due to division-by-zero conditions during flow-rate computation. There were no negative infinite values detected. Infinite values for these flows were not discarded or replaced by statistical imputation; instead, each was replaced by the maximum finite value calculated from the same feature in the training partition. This approach preserves the corresponding traffic observations at an extremely high transmission rate without causing numerical instability during Min–Max normalization. The maximum-finite-value replacement preserved all 611 affected network flows while preventing the positive infinite values from propagating into the normalization and model-training stages. After replacement, the affected feature values remained finite and were successfully mapped to the [0,1] range by Min–Max normalization, without requiring removal of the corresponding traffic observations. In addition, computing replacement values from the training partition does not leak information into the validation and independent test sets, thus maintaining a valid experimental evaluation.

**Table 3:** Non-finite value handling during data preprocessing pipeline.

Item	Value
Total feature values inspected	25,064,640
Missing values	0
Positive infinite values	742
Negative infinite values	0
Network flows containing at least one infinite value	611
Affected feature columns	Rate, Srate, Drate
Replacement strategy	Maximum finite value computed from the corresponding training-set feature
Normalization	Min–Max scaling [0,1]

The normalized feature vector was subsequently reshaped into a one-dimensional representation for convolutional processing, as shown in Equation (5):

$$x\in {\mathbb{R}}^{45\times 1}$$ (5)

The one-dimensional convolutional neural network can capture local interactions between adjacent features, while the MHSA process can capture long-range interactions between different features.

The order of the input feature vector is fixed and deterministic across all experiments, consistent with the order of features in the CICIoMT2024 dataset. The features are not arbitrary but rather are grouped in semantically related clusters based on their network characteristics, as summarized in Table 4. By placing attributes that are statistically relevant to each other next to each other, this organization allows the Conv1D layers to capture local interactions among the adjacent traffic features before the MHSA module captures long-range interactions between the whole feature representation.

Algorithm 1: MQTT attack dataset construction.
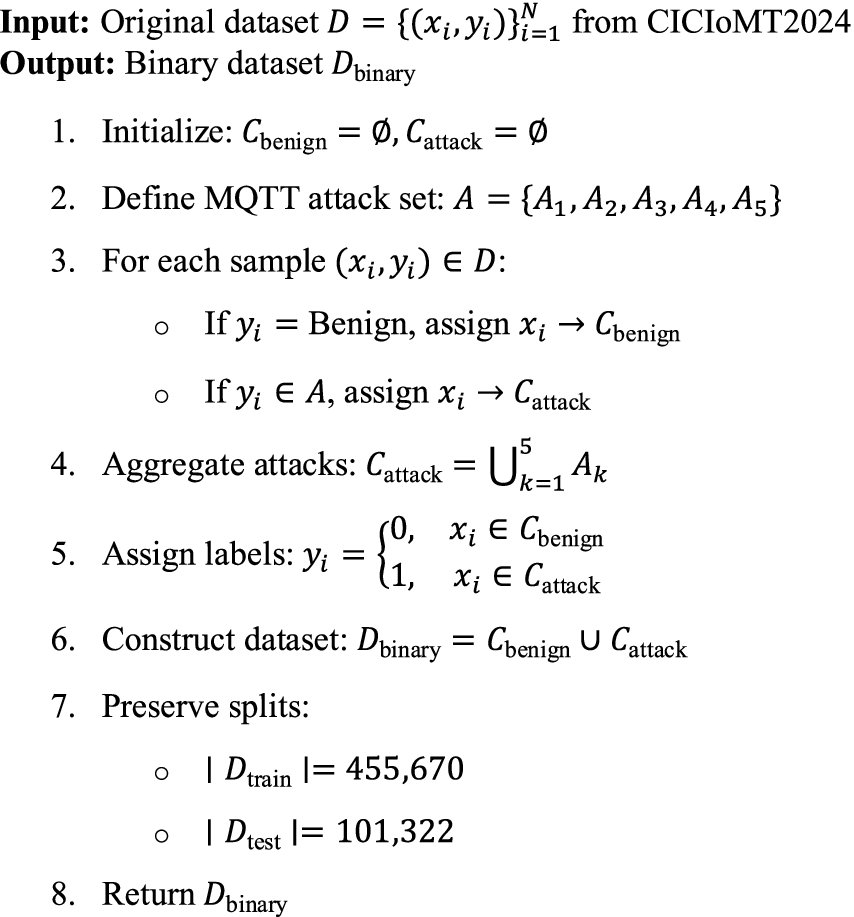

**Table 4:** Features used for train, test and validation of the CNN–MHSA.

Group	Features
Flow-level statistics	Flow Duration, Header Length, Protocol Type, Duration, Rate, Srate, Drate
TCP control flag features	fin_flag_number, syn_flag_number, rst_flag_number, psh_flag_number, ack_flag_number, ece_flag_number, cwr_flag_number, ack_count, syn_count, fin_count, urg_count, rst_count
Protocol-specific indicators	HTTP, HTTPS, DNS, Telnet, SMTP, SSH, IRC, TCP, UDP, DHCP, ARP, ICMP, IPv, LLC
Statistical traffic descriptors	Tot sum, Min, Max, AVG, Std, Tot size, IAT, Number, Magnitude, Radius, Covariance, Variance

### 3.3 Proposed deep learning model

This research presents a hybrid deep learning methodology that incorporates one-dimensional convolutional neural networks and multi-head self-attention for detecting protocol-specific intrusions in IoMT data. Local feature interactions and global dependencies are important aspects of network traffic characteristics that the CNN-MHSA system captures to identify subtle irregularities in the MQTT communication stream, such as illegal access attempts and data exfiltration. Figure 2 and Table 5 illustrate the overall architecture and layer-by-layer implementation of the proposed CNN–MHSA framework. Figure 2 presents the computational workflow, whereas Table 5 summarizes the implementation details, including tensor dimensions, architectural parameters, and layer configurations.

**Figure 2:**
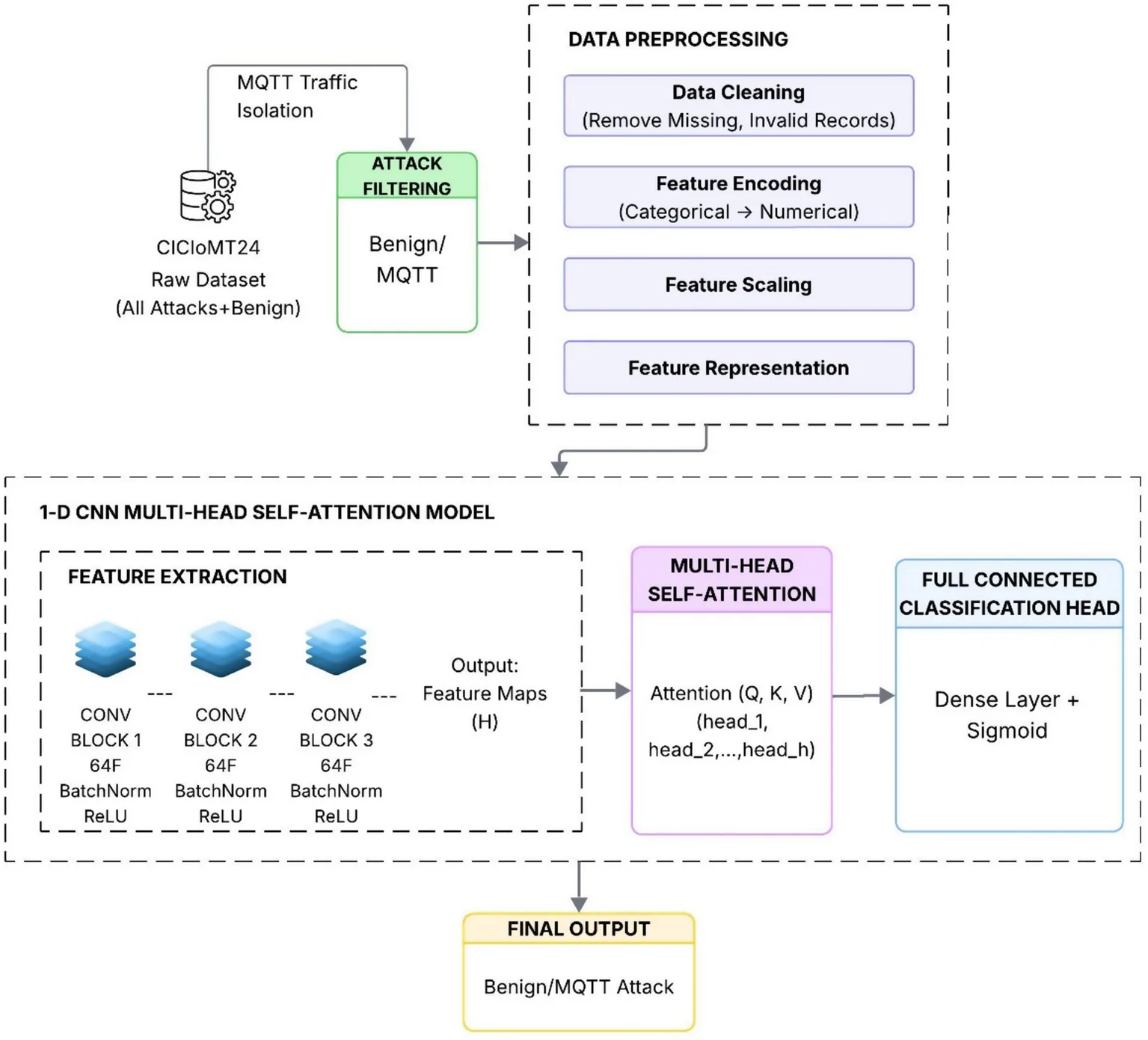
Architecture of the proposed 1D CNN–Multi-Head Self-Attention Model.

**Table 5:** Layer-wise configuration of the proposed CNN–MHSA framework.

Layer	Output tensor	Filters/Units	Kernel size	Stride	Padding	Pooling	Activation	Attention configuration	Parameters^*^	Purpose
Input	(45 × 1)	–	–	–	–	None	–	–	0	Normalized network-flow feature vector containing 45 statistical features
Conv1D-1	(45 × 64)	64 filters	3	1	Same	None	ReLU	–	256	Initial local feature extraction
Batch normalization	(45 × 64)	64 features	–	–	–	None	–	–	256	Normalizes intermediate feature maps
Dropout	(45 × 64)	–	–	–	–	None	–	–	0	Dropout regularization (rate = 0.3)
Conv1D-2	(45 × 64)	64 filters	3	1	Same	None	ReLU	–	12,352	Hierarchical local feature extraction
Batch normalization	(45 × 64)	64 features	–	–	–	None	–	–	256	Normalizes intermediate feature maps
Dropout	(45 × 64)	–	–	–	–	None	–	–	0	Dropout regularization (rate = 0.3)
Conv1D-3	(45 × 64)	64 filters	3	1	Same	None	ReLU	–	12,352	Hierarchical local feature extraction
Batch normalization	(45 × 64)	64 features	–	–	–	None	–	–	256	Normalizes intermediate feature maps
Dropout	(45 × 64)	–	–	–	–	None	–	–	0	Dropout regularization (rate = 0.3)
Multi-head self-attention	(45 × 64)	7 heads	–	–	–	None	–	*q* = 64, *k* = 64, *v* = 64, Heads = 7	283,648	Captures long-range dependencies among convolutional feature maps
Dense	(256)	256 Units	–	–	–	None	ReLU	–	16,640	High-level feature representation
Output dense	(1)	1 Unit	–	–	–	None	Sigmoid	–	257	Binary classification (Benign/MQTT attack)

Suppose that the normalized feature vector of a network flow is $x\in {\mathbb{R}}^{45\times 1}$ . The model input undergoes sequential convolutional feature extraction, attention-based representation learning, and classification.

This design is well suited for MQTT traffic because local statistical patterns coexist with long-range feature dependencies that may not be adequately captured by standalone convolutional or attention-based architectures.

#### 3.3.1 Convolutional feature extraction

Initially, the model utilizes a sequence of one-dimensional convolutional layers to extract distinctive local feature patterns from the network flow. The formula of the convolutional layer is given by Equation 6:

$$h_{i}=\sigma (\sum_{k=0}^{K-1}w_{k}\cdot x_{i+k}+b)$$ (6)

In this context, x denotes the input feature vector, $w_{k}$ indicates kernel weights, b indicates the bias term, K signifies the kernel size, and σ(·) refers to the nonlinear activation function.

The convolutional feature extractor comprised a three-layer stack of Conv1D, each employing 64 filters with a kernel size of three and ReLU activation functions. This enables hierarchical extraction of local feature patterns across successive convolutional layers, progressively building richer representations of the input traffic. Batch normalization was applied after each Conv1D layer, followed by ReLU activation, and a dropout rate of 0.3 was subsequently employed within each convolutional block to stabilize training and mitigate overfitting. The resulting feature maps capture complex correlations among traffic statistics, packet behavior, and protocol-level attributes within the network flow. The adopted feature ordering further enables adjacent convolutional kernels to exploit local relationships within each semantic feature group, while the subsequent MHSA module captures complementary long-range dependencies across different feature groups.

#### 3.3.2 Multi-head self-attention mechanism

Although convolutional layers are useful for capturing local relationships of features, network traffic also has long-range relationships of features in intrusion patterns of network traffic, which may be essential for adequately identifying intricate attack scenarios. Therefore, an MHSA mechanism is inserted after the convolutional feature extraction stage to capture these long-range relationships, which will improve the system’s capacity to identify complex attack scenarios.

The feature maps generated by the final convolutional layer are directly supplied to the multi-head self-attention module. Let the resulting convolutional representation be denoted by H , from which the query, key, and value matrices are determined as delineated in Equation 7:

$$Q=HW_{Q},K=HW_{K},V=HW_{V}$$ (7)

where $W_{Q},W_{K},W_{V}$ are learnable projection matrices.

The attention output, calculated utilizing scaled dot-product attention, is delineated in Equation 8:

$$\text{Attention}(Q,K,V)=\text{softmax}(\frac{QK^{T}}{\sqrt{d_{k}}})V$$ (8)

where $d_{k}$ denotes the dimensionality of the key vectors.

To augment the representation capability, multiple attention heads are utilized, as shown in Equation 9:

$$\text{MHSA}(Q,K,V)=\text{Concat}(\mathrm{hea}d_{1},\mathrm{hea}d_{2},\ldots ,\mathrm{hea}d_{h})W_{O}$$ (9)

where h=7 describes the quantity of attention heads and $W_{O}$ is the output projection matrix. The number of attention heads ( h=7 ) was selected based on validation-driven hyperparameter tuning, where it achieved the highest validation AUC compared to candidate values {4, 6, 7, 8, 10}, providing an effective balance between representational capacity and computational efficiency.

#### 3.3.3 Classification layer

Two fully connected layers process the attention-enhanced feature representation. The first dense layer learns a high-level latent representation, while the final sigmoid-activated output layer produces the probability score p(y=1∣x) .

The probability was computed via the sigmoid activation function, as specified in Equation 10:

$$p(y=1|x)=\sigma (z)=\frac{1}{1+e^{-z}}$$ (10)

Here z represents the output of the final dense layer.

Model parameters are tuned via the AdamW optimizer with an initial learning rate of $10^{-3}$ . The training goal was to reduce the binary cross-entropy loss, as expressed in Equation 11:

$$L=-\frac{1}{N}\sum_{i=1}^{N}[y_{i}\log(p_{i})+(1-y_{i})\log(1-p_{i})]$$ (11)

where N denotes the quantity of training data and $p_{i}$ signifies the predicted probability for the sample i .

Algorithm 2 briefly outlines the complete training process for the CNN-MHSA-based intrusion detection approach, including the mathematical steps used to train the model. For reproducibility, the detailed layer-by-layer implementation of the proposed CNN–MHSA architecture is presented in Table 5, including tensor dimensions, architectural settings, attention configuration, and parameter allocation.

Algorithm 2: CNN–MHSA-based intrusion detection model.
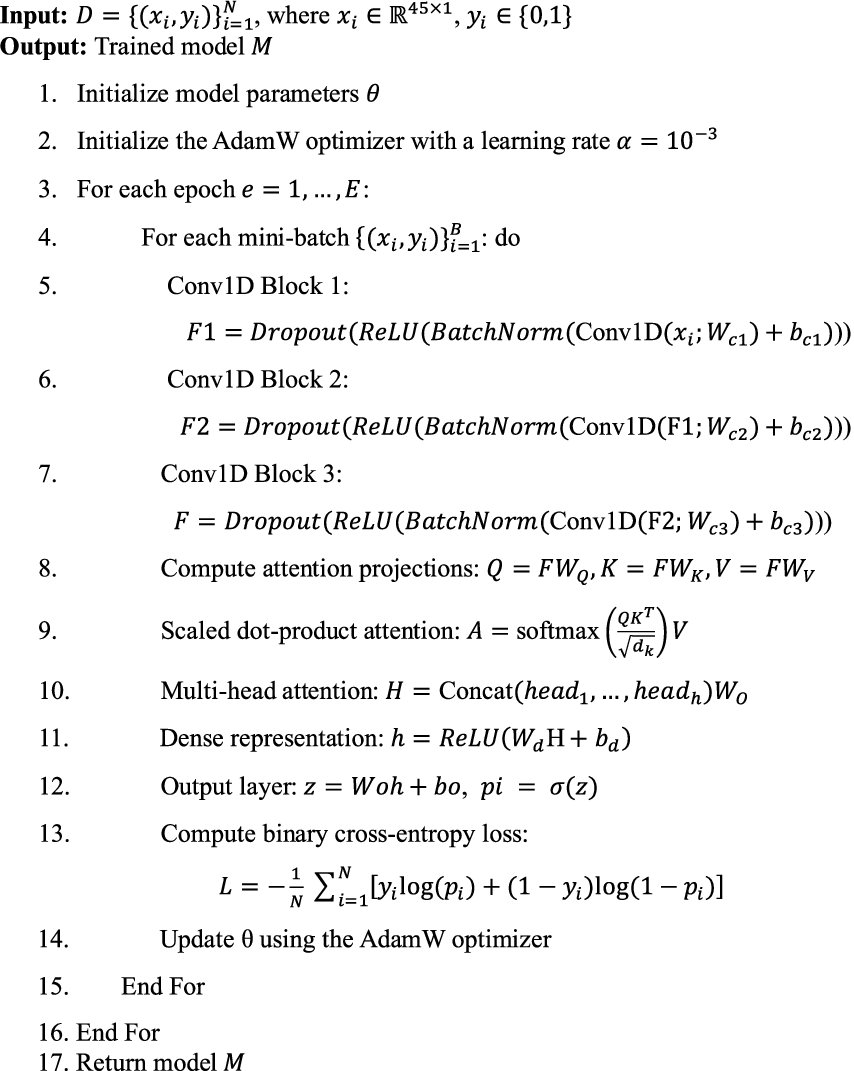

### 3.4 Training strategy and experimental configuration

The proposed framework was executed utilizing Python 3.10.12 and TensorFlow 2.15. Model training was performed on a high-performance computing machine using an Intel Xeon E5-2686v4 CPU, 256 GB of RAM, and an NVIDIA Tesla V100 GPU featuring 32 GB of memory. To assess deployment-related computational characteristics, inference profiling was performed separately on a CPU-only platform comprising a single-core virtualized Intel Xeon processor operating at 2.10 GHz, 3.9 GB of RAM, and no GPU acceleration.

In order to account for a moderate class imbalance in the training data (57.7% attack, 42.3% benign samples), the class-weighting method was applied to the optimization of the model using the standard balanced class-weighting formulation, as shown in Equation (12):

w_c=N/(K×N_c) (12)

where N denotes the overall count of training samples, K represents the total number of classes, and N_c corresponds to the number of samples belonging to class c . According to the reported composition of the training partition (1,92,732 benign samples and 2,62,938 attack samples), the resulting class weights were 1.18 for the benign class and 0.87 for the attack class. This weighting penalizes minority-class errors more heavily and encourages balanced learning for both the minority class and the majority class.

#### 3.4.1 Threshold optimization

No standard value was used for the classification threshold of 0.50. However, a validation-based approach was used to set an appropriate decision threshold for the proposed intrusion detection model. In model development, various threshold values were systematically tested on an internal validation partition to explore the balance between precision and recall. The threshold value was set to 0.40, which maximized the validation F1-score and provided a satisfactory balance between detection sensitivity and false alarm control. In this study, this threshold is a viable option for a moderate class imbalance (57.7% attack and 42.3% benign samples in the training set).

The chosen threshold provides a balance between detection sensitivity and false alarm control while maintaining high sensitivity. While a false negative is a more important issue in IoMT applications due to the possibility of compromising patient safety and service availability, tuning for higher recall can significantly increase the probability of false alarms and decrease operational usability. Hence, the F1 score was used as a threshold selection criterion, which considers both precision and recall. The selected threshold achieved a recall of 99.58% and a false-negative rate of 0.42% (268 misclassified attack instances out of 63,715 MQTT attack samples) on the independent test set, which demonstrated a conservative bias against missed detections, consistent with the asymmetric cost of false negatives in safety-critical environments for IoMT environments. Explicit cost-sensitive threshold selection strategies such as Fβ-based optimization (*β* > 1) and cost-matrix-driven approaches will be studied in future work to further formalize the trade-off between missed detections and false alarms. The complete training and architectural hyperparameters used for model development are summarized in Table 6. These hyperparameters include the optimization strategy, convolutional configuration, attention-module settings, dense-layer configuration, initialization strategy, and training parameters used throughout all experiments.

**Table 6:** Model training and architecture hyperparameters.

Hyperparameter	Value
Optimizer	AdamW
Initial learning rate	0.001
Weight decay	0.0001
Loss function	Binary cross-entropy
Batch size	256
Maximum training epochs	50
Early stopping patience	5 epochs
Classification threshold	0.40
CNN blocks	3
Number of CNN filters	64
Convolution kernel size	3
Convolution stride	1
Padding	Same
Activation function	ReLU
Batch normalization	Applied after each Conv1D layer
Dropout rate	0.30
Number of attention heads	7
Attention key dimension	64
Attention value dimension	64
Hidden dense layer	256 units
Output layer	1 unit (Sigmoid)
Weight initialization	Glorot uniform
Random seed	42

The architectural hyperparameters were selected according to the characteristics of the input representation and the performance on the validation set. With the low dimensionality of the input feature space, three convolutional layers were used to trade off representational capacity against overfitting risk. The kernel size of 3 was selected because it reduced the number of parameters and captured local interactions between the neighboring flow statistics. The decoupled weight decay mechanism employed by the AdamW optimizer provides superior regularization properties compared with L2-penalized optimization, resulting in improved generalization on datasets with a moderate degree of imbalance.

To ensure reproducibility of data partitioning, parameter initialization, and model training, the primary experiments were carried out with the same fixed random seed of 42. To assess the reliability of the proposed framework with respect to random variations in training, further experiments were conducted using multiple independent random seeds.

### 3.5 Hyperparameter optimization

Structured validation-driven search was performed to identify the principal hyperparameters listed in Table 6. The internal validation set was used to evaluate candidate values for various parameters. For architectural consistency, the convolution parameters, stride, weight initialization, dense-layer configuration, and attention dimensions were held constant across all experiments, ensuring the architecture remained consistent throughout the hyperparameter optimization. Based on the validation AUC, the candidate set for the number of attention heads (h) was {4, 6, 7, 8, 10}, with 7 as the best validation configuration. The initial learning rate was chosen from {10^−2^, 10^−3^, 10^−4^}, and the dropout rate was tested across {0.20, 0.30, 0.50}. To keep the network lightweight, the number of convolutional filters (64) and the size of the convolutional kernel (3) were fixed, following the design guidelines for one-dimensional convolutional networks used for tabular network traffic data. The model with the best validation loss was saved after early stopping at five epochs and used for the final evaluation on the test set.

## 4 Results and performance evaluation

### 4.1 Experimental setup and evaluation protocol

The effectiveness of the CNN-MHSA system for intrusion detection was evaluated using the established train-test partition from the CICIoMT2024 dataset. The dataset comprises 455,670 training samples (81.8%) and 101,322 test samples (18.2%). The independent test set was excluded from model development to ensure the original benchmark protocol remains reliable and to prevent data leakage. The predefined training and testing partitions provided by the CICIoMT2024 dataset are from different PCAP capture files so that network flows in the training, validation, and testing partitions are not shared across the same PCAP capture file.

In developing the model, a 20% stratified subset of the predefined training partition was reserved for internal validation hyperparameter tuning and threshold optimization, and 80% of the partition was reserved for model training. The validation set consisted of 91,134 samples (38,546 benign samples and 52,588 MQTT attack samples), while the training set contained 3,64,536 samples (1,54,186 benign samples and 2,10,350 MQTT attack samples). The original class distribution was maintained in all data partitions using stratified sampling. The validation set is used solely for hyperparameter selection and threshold determination and was not subjected to the final test set evaluation process.

The final version was assessed solely on the independent test set, which included 101,322 network flow instances, including 37,607 benign samples and 63,715 MQTT attack samples. The separation between the training and independent test sets ensures that the reported outcomes accurately represent the ability to generalize the proposed approach to new IoMT traffic. The model produces a probability score representing the likelihood of malicious activity. During evaluation, the validation-optimized classification threshold determined in Section 3.4.1 was fixed and applied to the independent test set.

All of the following standard performance metrics, including accuracy, precision, recall, F1-score, and the area under the receiver operating characteristic curve (AUC-ROC), have been used to assess detection performance. Table 7 presents the mathematical definition and description of the evaluation measures used in this investigation.

**Table 7:** Performance evaluation metrics.

Metric	Mathematical definition	Description
Accuracy	$\text{Accuracy}=\frac{\mathrm{TP}+\mathrm{TN}}{\mathrm{TP}+\mathrm{TN}+\mathrm{FP}+\mathrm{FN}}$	Evaluates the total ratio of accurately categorized traffic cases, encompassing both benign and attack samples
Precision	$\text{Precision}=\frac{\mathrm{TP}}{\mathrm{TP}+\mathrm{FP}}$	Assesses the dependability of attack predictions by calculating the ratio of correctly identified assaults to the total expected attacks
Recall (Detection rate)	$\text{Recall}=\frac{\mathrm{TP}}{\mathrm{TP}+\mathrm{FN}}$	Indicates the capacity to reliably recognize malicious traffic, reflecting the proportion of successfully identified assaults
F1-score	$F1=\frac{2\times \text{Precision}\times \text{Recall}}{\text{Precision}+\text{Recall}}$	An in-depth evaluation of precision and recall, especially beneficial for analyzing classification efficacy in situations of class imbalance
AUC-ROC	$\mathrm{AUC}=\int_{0}^{1}\mathrm{TPR}(u)\mathrm{du}$	Evaluates the ability to distinguish among benign and malicious traffic at many classification tiers

### 4.2 Classification performance of the proposed model

Evaluation of the CNN-MHSA framework is quantified by classification accuracy on the independent test set of benign and MQTT attack traffic. During test-set evaluation, the CNN-MHSA model achieved 99.40% accuracy, demonstrating the model’s high reliability. A 99.47% precision score means the system has a very low false-positive rate, indicating that any traffic detected as malicious is likely to be classified correctly. A recall rate of 99.58% indicates that the suggested approach effectively identifies most MQTT attacks, which reduces the chance of undetected intrusions. The F1-score of 99.52% is an indicator of an optimal balance between precision and recall, implying that the framework has a high detection rate and minimal incidence of false positives. The AUC-ROC value of 0.9998 shows that the framework is highly capable of differentiating benign and attack traffic across a wide range of classification thresholds with high accuracy. Table 8 highlights that the results validate the efficacy of the CNN-MHSA mechanism for learning discriminative feature representations of traffic on the IoMT network.

**Table 8:** Evaluation of the proposed CNN–MHSA model’s performance.

Metric	Value
Accuracy (%)	99.40
Precision (%)	99.47
Recall (%)	99.58
F1-score (%)	99.52
AUC-ROC	0.9998

#### 4.2.1 Statistical confidence analysis

The 95% confidence intervals (CIs) were estimated for the main evaluation metrics to quantify the statistical reliability of the classification performance reported. Confidence intervals for accuracy, precision, recall, specificity, false positive rate (FPR), and false negative rate (FNR) were calculated as proportions based on the confusion matrix and were estimated by the Wilson score interval, as this interval is more accurate than the normal approximation interval in terms of coverage performance, especially when proportions are near 0 or 1. The F1-score is a non-linear metric, so the observed test-set confusion matrix was multinomial bootstrap resampled (10,000 bootstrap samples) to estimate the F1-score confidence interval. The confidence intervals obtained are listed in Table 9.

**Table 9:** Statistical confidence intervals for the proposed CNN-MHSA model.

Metric	Value (%)	95% CI lower	95% CI upper
Accuracy	99.40	99.35	99.45
Precision	99.47	99.41	99.52
Recall	99.58	99.53	99.63
F1-score	99.52	99.49	99.56
Specificity	99.10	99.00	99.19
False negative rate	0.42	0.37	0.47
False positive rate	0.90	0.81	1.00

All of the confidence intervals are within a 0.20 percentage point width and remain narrow. This result is due to the large test set used, which is independent of the other test sets, and it indicates that the reported performance estimates have a small sampling uncertainty, meaning they are statistically stable under the experimental protocol employed. The high precision and recall, along with their small confidence intervals, further show that the proposed CNN-MHSA framework effectively distinguishes benign traffic from MQTT attack traffic.

#### 4.2.2 Robustness across multiple random seeds

The proposed CNN-MHSA framework was evaluated independently five times with different random seeds, which were used to initialize the parameters and shuffle the training data, as well as stochastic optimization, while keeping the same independent test set for each evaluation. The performance metrics that result from this are summarized in Table 10.

**Table 10:** Performance of the proposed CNN-MHSA framework across five independent training runs.

Random seed	Accuracy (%)	Precision (%)	Recall (%)	F1-score (%)	AUC
0	99.31	99.38	99.44	99.41	0.9996
1	99.40	99.47	99.58	99.52	0.9998
42	99.44	99.51	99.61	99.56	0.9998
123	99.28	99.35	99.49	99.42	0.9995
2024	99.37	99.44	99.53	99.48	0.9997
Mean ± SD	99.36 ± 0.06	99.43 ± 0.06	99.53 ± 0.07	99.48 ± 0.06	0.9997 ± 0.0001

The proposed CNN-MHSA framework showed very low variation for all the main evaluation metrics across all five independent training runs, with standard deviations being less than 0.10 percentage points. This small variation in accuracy, precision, recall, F1-score, and AUC indicates that the proposed architecture is robust to stochastic variations introduced by the initialization of the parameters and training dynamics. This result indicates that the reported performance is reproducible and not just a favorable training session or a random seed.

### 4.3 Confusion matrix analysis

The extensive evaluation of the CNN-MHSA model’s classification for efficacy is provided by the confusion matrix in Figure 3. Figure 3 shows a breakdown of its predictions for benign and MQTT attack categories. It achieved a correct classification of 37,267 benign instances (true negatives) and 340 incorrect classifications of benign instances (false positives). Attack instances were also accurately detected (true positives: 63,447), and very few attack samples were wrongly classified as benign (false negatives: 268).

**Figure 3:**
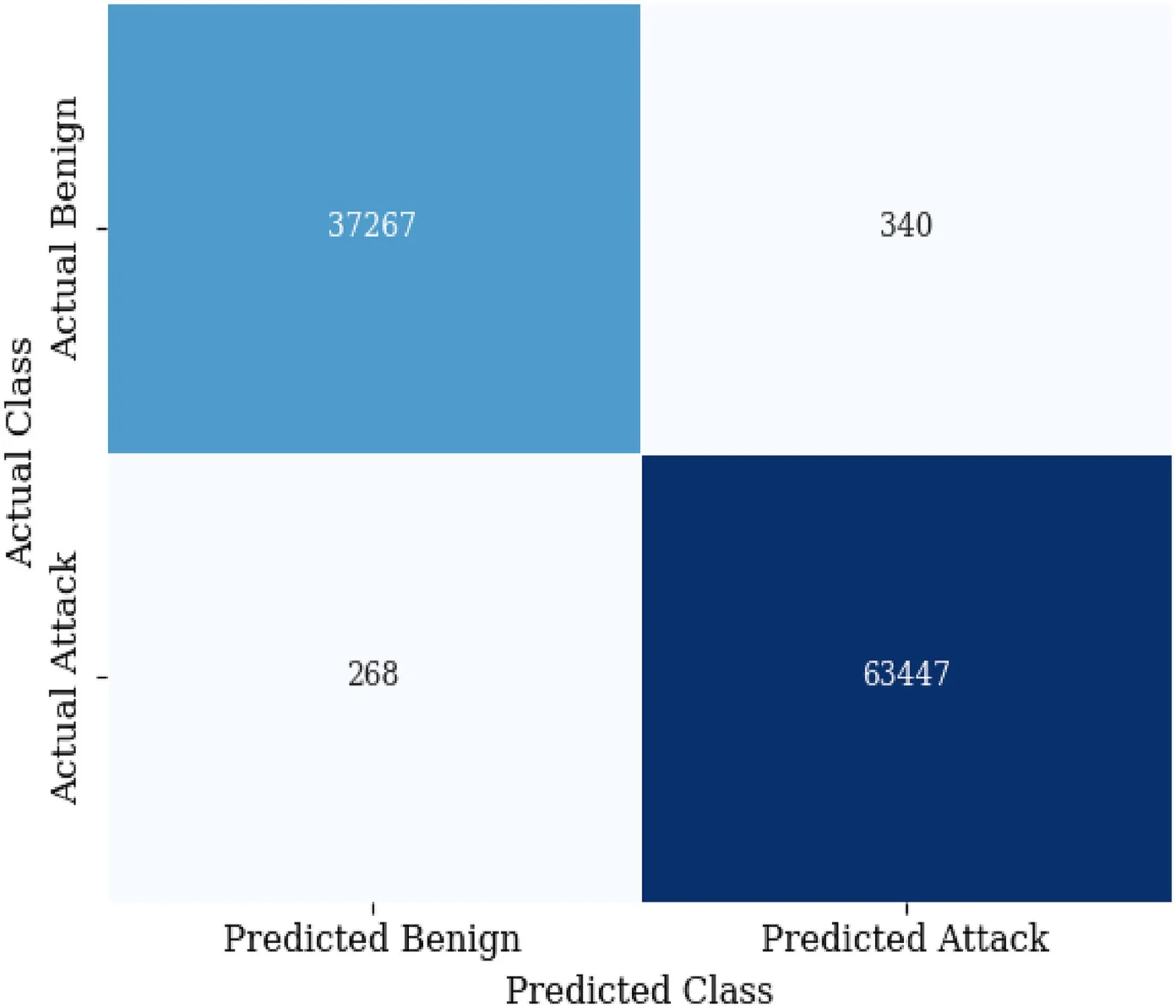
Confusion matrix of the proposed CNN–MHSA model.

The results demonstrate that the proposed framework has a very low false-negative rate (0.42%) and a low false-positive rate (0.90%), indicating high detection sensitivity and dependability in distinguishing malicious activities and normal traffic. The low rate of false negatives is particularly important in IoMT security, as undetected intrusions could have serious consequences such as unauthorized access and disruption of required medical services. The false-positive rate is also low; the system is stable and not generating too many false alarms. The results of the confusion matrix analysis show that the proposed CNN-MHSA approach is able to achieve a reasonable balance between detection and false alarm control, making it suitable for accurate and stable MQTT intrusion detection in IoMT networks.

### 4.4 ROC curve and discriminative performance analysis

The ROC curve illustrates the system efficacy in differentiating between benign IoMT traffic and malicious traffic via MQTT across various decision criteria. Figure 4 illustrates that the ROC curve predominantly occupies the upper left quadrant of the feature space, signifying the system’s capacity to attain a high true positive rate while maintaining a comparatively low false positive rate across a broad spectrum of thresholds. A large diagonal reference line, indicative of random categorization, also indicates strong class separability of the obtained feature representations.

**Figure 4:**
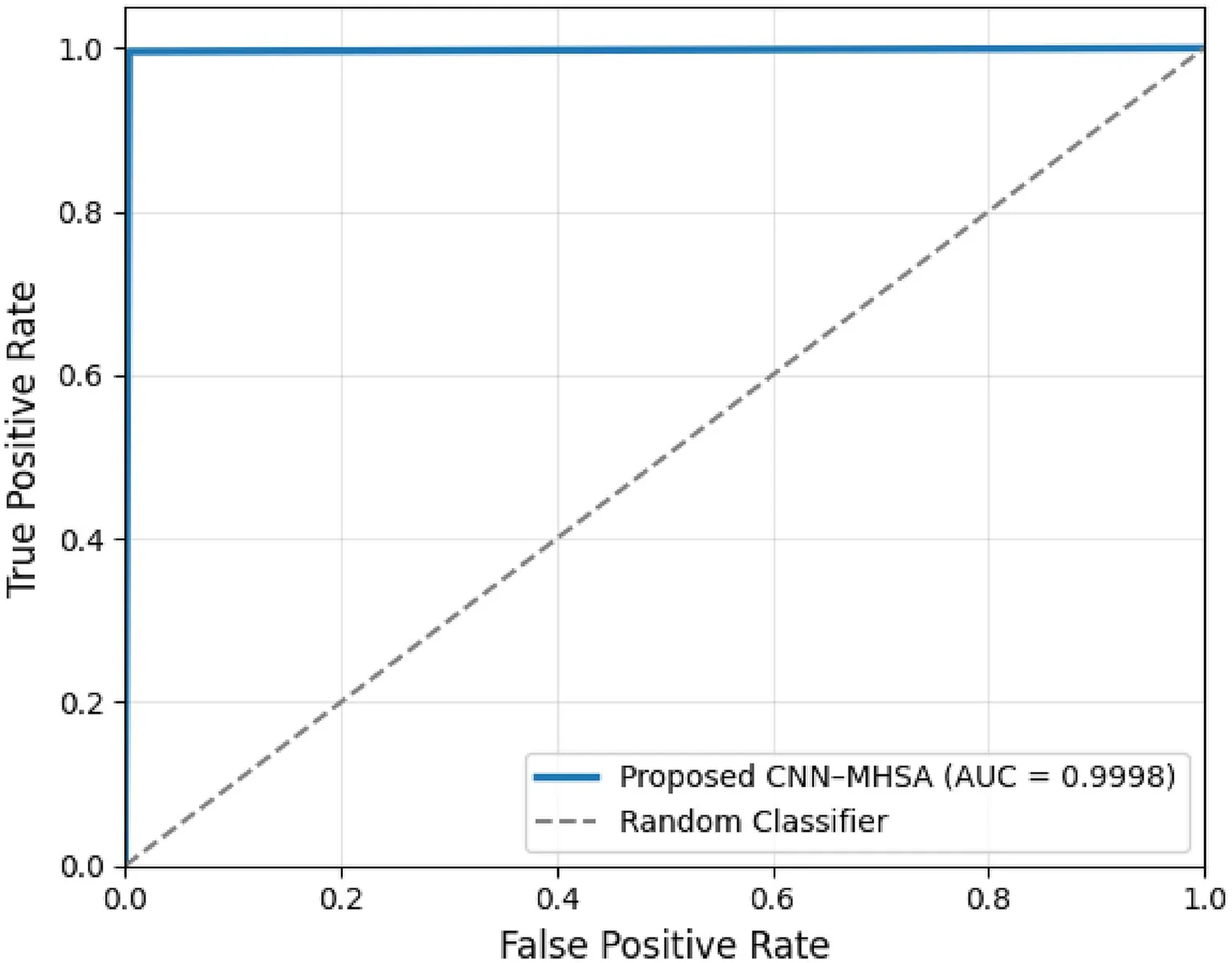
ROC curve of the proposed CNN–MHSA model.

The AUC-ROC was 0.9998, indicating nearly flawless differentiation between benign and attack traffic. This additional representation learning enables the model to recognize minute differences in network traffic that distinguish regular communication patterns from MQTT-based attack patterns, which is crucial for improving the system to detect possible threats in IoMT traffic.

### 4.5 Comparison with existing methods

The proposed CNN-MHSA framework is contrasted with representative machine learning, hybrid deep learning, attention-based, transformer-based, and graph neural network intrusion detection approaches reported in recent literature to evaluate its effectiveness and position it relative to the current state of the art. Because the selected studies employ different datasets, classification settings (binary, multiclass, and protocol-specific), and experimental protocols, the comparison is intended to provide qualitative and quantitative context rather than a direct benchmark. To further strengthen the comparison, Table 11 additionally includes recent intrusion detection studies evaluated on the CICIoMT2024 dataset.

**Table 11:** Comparison of the proposed method with representative state-of-the-art intrusion detection approaches.

Ref	Dataset	Method	Accuracy (%)	Precision (%)	Recall (%)	F1-score (%)	AUC
Aqachtoul et al. (2025)	MQTTEEB	DT + GRU	87	87	87	86	N/R
Zeghida et al. (2023)	MQTT dataset	Ensemble ML	95.43	N/R	N/R	95.42	N/R
Boppana and Bagade (2023)	MQTT-IoT-IDS2020	OCSVM	96.90	97.70	97.60	97.60	N/R
Putro et al. (2025)	RT-IoT2022	KNN	98.87	99.07	99.67	99.37	N/R
Prajisha and Vasudevan (2022)	MC-IoT/MQTTset/MQTT-IoT-IDS2020	ECSSA-LightGBM	98.91	98.80	98.36	97.16	N/R
Zeghida et al. (2025b)	MQTTset	CNN–LSTM	99	100	100	99	N/R
Zuhairi et al. (2024)	MQTTset	Decision tree	99.27	N/R	N/R	99.26	N/R
Mohammadi et al. (2024)	CICIoMT2024	CNN	99	99	99	99	N/R
Sohail et al. (2024)	CICIoMT2024	XGBoost	95.01	N/R	N/R	N/R	N/R
Mondal et al. (2026)	CIC-BCCC-NRC-IoMT2024	QAE-IDS	96.22	96.36	96.22	96.26	0.997
Ullah et al. (2023a)	Edge-IIoTset	MAGRU-IDS	99.99	99.99	99.99	99.99	N/R
Ullah et al. (2023b)	MQTT-IoT-IDS2020	TNN-IDS (Transformer)	99.99	99.99	99.99	99.99	N/R
Akuthota and Bhargava (2025)	NSL-KDD/UNSW-NB15	Transformer IDS	99.97	99.97	99.97	99.97	N/R
Reka et al. (2024)	MANET simulation	MSA-GCNN	97.12	N/R	N/R	N/R	N/R
Proposed (Ours)	CICIoMT2024	CNN-MHSA	99.40	99.47	99.58	99.52	0.9998

N/R denotes that the corresponding metric was not reported in the original publication, which includes representative intrusion detection methods evaluated on multiple benchmark datasets together with recent studies using the CICIoMT2024 dataset.

As summarized in Table 11, conventional machine learning and ensemble-based methods have demonstrated competitive detection performance for MQTT intrusion detection but generally rely on feature engineering and dataset-specific optimization. Aqachtoul et al. (2025) developed a hybrid DT-GRU method for the MQTTEEB dataset and reported 87% accuracy, whereas Zeghida et al. (2023) achieved 95.43% accuracy using an ensemble machine learning framework on the MQTT dataset. Boppana and Bagade (2023) employed a one-class SVM for anomaly detection on the MQTT-IoT-IDS2020 dataset, achieving 96.90% accuracy. Similarly, Putro et al. (2025) showed the efficacy of instance-based learning using KNN on the RT-IoT2022 dataset, while Prajisha and Vasudevan (2022) improved intrusion detection performance by combining optimization-based feature selection with the ECSSA-LightGBM ensemble model. Zeghida et al. (2025b) further showed that a hybrid CNN-LSTM architecture can effectively capture temporal characteristics of MQTT traffic, achieving 99.00% accuracy on the MQTTset dataset, whereas Zuhairi et al. (2024) demonstrated that a decision tree classifier can achieve competitive performance (99.27%) under controlled MQTT traffic conditions.

More recently, several studies have evaluated intrusion detection models using the CICIoMT2024 dataset. Mohammadi et al. (2024) proposed a CNN-based intrusion detection framework and reported approximately 99% accuracy across binary, six-class, and multiclass classification tasks. Sohail et al. (2024) investigated explainable boosting ensemble methods on the CICIoMT2024 dataset using the XGBoost model and achieved 95.01% accuracy for multiclass intrusion detection. Mondal et al. (2026) proposed a hierarchical Quantized Autonomous Edge IDS combining lightweight 1D-CNN and XGBoost, achieving 96.22% multiclass accuracy on the CIC-BCCC-NRC-IoMT2024 dataset while emphasizing deployment efficiency and explainability. These studies demonstrate the increasing adoption of the CICIoMT2024 dataset for evaluating modern IoMT intrusion detection frameworks and provide additional context for assessing the proposed model against recent research using the same benchmark dataset.

Recent studies have increasingly adopted attention-driven architectures to improve intrusion detection performance. MAGRU-IDS integrates multi-head attention with gated recurrent units to enhance minority-class recognition in IIoT traffic (Ullah et al., 2023a), while TNN-IDS utilizes a transformer architecture to capture long-range feature dependencies in MQTT network traffic (Ullah et al., 2023b). Likewise, Akuthota and Bhargava (2025) proposed a transformer-based intrusion detection framework evaluated on the NSL-KDD and UNSW-NB15 datasets, demonstrating strong generalization across conventional benchmark datasets. Reka et al. (2024) introduced the MSA-GCNN model, which combines graph convolutional networks with multi-head self-attention to exploit structural relationships in MANET traffic. These approaches demonstrate the effectiveness of attention mechanisms in improving feature representation; however, they are primarily designed for temporal sequence learning, graph-based representation learning, or general-purpose intrusion detection and are evaluated on datasets that differ substantially from contemporary IoMT environments. Unlike these architectures, which individually emphasize recurrent sequence modeling, pure transformer encoding, or graph-structured feature learning, the proposed CNN-MHSA framework explicitly combines lightweight convolutional feature extraction with multi-head self-attention within a unified architecture, enabling simultaneous learning of local statistical patterns and long-range feature dependencies in protocol-specific MQTT traffic.

In contrast, the proposed framework is specifically designed for protocol-specific MQTT intrusion detection using the recent CICIoMT2024 dataset. Rather than relying solely on recurrent, transformer, or graph-based representations, the proposed architecture first employs lightweight one-dimensional convolution to learn hierarchical local traffic patterns and then applies multi-head self-attention to capture long-range feature dependencies. This complementary design enables efficient learning of both local and global characteristics of MQTT network flows while maintaining a lightweight architecture suitable for IoMT environments. The suggested CNN-MHSA framework achieves 99.40% accuracy, 99.47% precision, 99.58% recall, 99.52% F1-score, and an AUC of 0.9998, according to experimental data. Compared with existing CNN, ensemble learning, transformer-based, recurrent attention-based, graph attention, and recent CICIoMT2024-based intrusion detection models, the proposed CNN-MHSA framework achieves competitive performance while employing a lightweight convolution-attention architecture specifically designed for protocol-specific MQTT intrusion detection. Furthermore, the ablation study, computational efficiency analysis, and cross-dataset validation presented in this work provide additional evidence supporting the reliability and generalization potential of the proposed framework beyond conventional performance comparisons.

### 4.6 Ablation study

An ablation study was conducted to determine the influence of different structural elements, utilizing three model configurations assessed on the same independent MQTT test set. The first configuration consisted of an MHSA-only variant, in which the multi-head self-attention mechanism was applied directly to the normalized input feature representation without preceding convolutional feature extraction. The second configuration comprised a CNN-only variant containing the three-layer one-dimensional convolutional backbone and classification head without the attention module. The third configuration corresponded to the complete CNN-MHSA framework described in Section 3.3.

All model variants were trained using the same training-validation partition, class weighting strategy, optimization parameters, and five independent random seeds (0, 1, 42, 123, and 2024). For each architecture, the stated outcomes correspond to the mean performance across the five independent runs. Consistent with the training procedure described in Section 3.4.1, the classification threshold for each model variant was independently optimized by maximizing the validation F1-score using its respective validation predictions and subsequently fixed for evaluation on the independent test set.

As summarized in Table 12, each model variant was evaluated using its own validation-optimized decision threshold, and the reported results represent the mean performance across five independent training runs. Under these independently optimized operating points, the MHSA-only configuration achieved a mean accuracy of 97.45% and a mean AUC of 0.9951, indicating that attention mechanisms operating directly on raw flow features are insufficient to fully characterize MQTT attack patterns. The CNN-only configuration improved the mean accuracy to 98.89% and the mean AUC to 0.9989, demonstrating the effectiveness of convolutional feature extraction for learning local statistical characteristics within the 45-dimensional flow representation.

**Table 12:** Ablation study for the proposed CNN-MHSA framework.

Model	Threshold	Accuracy (Mean ± SD)	Precision (Mean ± SD)	Recall (Mean ± SD)	F1 (Mean ± SD)	AUC (Mean ± SD)
MHSA	0.45	97.45 ± 0.08	97.71 ± 0.09	97.98 ± 0.08	97.84 ± 0.08	0.9951 ± 0.0002
CNN	0.42	98.89 ± 0.07	99.03 ± 0.06	99.15 ± 0.06	99.09 ± 0.06	0.9989 ± 0.0001
CNN-MHSA (Proposed)	0.40	99.36 ± 0.06	99.43 ± 0.06	99.53 ± 0.07	99.48 ± 0.06	0.9997 ± 0.0001

The complete CNN-MHSA framework achieved the best mean performance, with an accuracy of 99.40%, an F1-score of 99.52%, and an AUC of 0.9998. These results demonstrate that convolutional feature extraction and multi-head self-attention provide complementary capabilities for modeling both local feature interactions and long-range dependencies relevant to MQTT intrusion detection.

Standard deviations computed over five independent training runs were found to be under 0.10 percentage points for all three variants of architecture, showing that the performance differences reported are statistically stable with respect to stochastic variation arising from parameter initialization due to parameter initialization and optimization. The consistently low variability adds further evidence that the superior performance of the proposed CNN–MHSA framework is reproducible and not due to a favorable random initialization. The decision thresholds determined using the three model variants were found independently, using the respective validation partition, and were then fixed during evaluation on the independent test partition so that the same test set was used for all three model variants but with different optimized decision thresholds, which gave a fair and unbiased comparison of the three model variants’ architectural configurations.

#### 4.6.1 Per-attack detection performance

Although the proposed CNN–MHSA framework was trained using a binary classification formulation in which all five MQTT attack categories were consolidated into a single attack class, the prediction outcomes on the independent test set were further analyzed separately according to the original MQTT attack categories. This additional analysis was conducted to determine whether the binary learning strategy masked weaker detection performance on less frequent attack types. As summarized in Table 13, the proposed CNN–MHSA framework consistently achieved high precision, recall, and F1-score across all five MQTT attack categories while maintaining a low number of false negatives. The results indicate that the proposed architecture generalizes effectively across different MQTT attack behaviors without exhibiting substantial degradation for less frequent attack categories.

**Table 13:** Per-attack detection performance of the proposed CNN–MHSA framework.

MQTT attack type	Precision (%)	Recall (%)	F1-score (%)	False negatives
MQTT-DDoS Connect Flood	99.93	99.90	99.91	41
MQTT-DoS Publish Flood	99.76	99.63	99.69	55
MQTT-DDoS Publish Flood	99.69	99.54	99.61	61
MQTT-DoS Connect Flood	99.42	99.18	99.30	50
MQTT-Malformed Data	98.91	98.52	98.71	61

#### 4.6.2 Statistical significance analysis

To statistically verify that the performance improvement achieved by the complete CNN-MHSA framework is not attributable to random sampling variation, a two-proportion z-test was performed to compare the proposed model with each ablated architecture using the classification results from the independent test set described in Section 4.1. Comparisons were conducted for both classification accuracy and attack recall using the corresponding test-set class distributions (63,715 MQTT attack samples and 37,607 benign samples). As summarized in Table 14, the proposed CNN-MHSA framework significantly outperformed both the CNN-only and MHSA-only configurations. Specifically, statistically significant improvements were observed over the CNN-only model for accuracy (*z* = 13.21, *p* < 0.001) and recall (*z* = 9.66, *p* < 0.001), while even larger improvements were obtained over the MHSA-only model for accuracy (*z* = 37.27, *p* < 0.001) and recall (*z* = 26.00, *p* < 0.001). These findings confirm that the performance gains achieved by integrating convolutional feature extraction with multi-head self-attention are statistically significant and provide quantitative evidence for the efficacy of the proposed hybrid architecture.

**Table 14:** Hypothesis testing results for the proposed CNN-MHSA framework and ablation variants.

Comparison	Metric	*z*-statistic	*p*-value	Significance
CNN-MHSA vs. CNN-only	Accuracy	13.21	<0.001	Significant
CNN-MHSA vs. CNN-only	Recall	9.66	<0.001	Significant
CNN-MHSA vs. MHSA-only	Accuracy	37.27	<0.001	Significant
CNN-MHSA vs. MHSA-only	Recall	26.00	<0.001	Significant

### 4.7 Computational efficiency analysis

The proposed CNN-MHSA model was profiled on a CPU-only platform to provide an estimate of the computational requirements for deployment, which differs from the GPU-based infrastructure used during model training. This provided a preliminary assessment of deployment-related computational requirements. The analysis was not based on actual deployment performance but on model complexity, memory footprint, inference latency, and throughput characteristics.

The proposed approach contains 3,26,273 parameters: 3,25,889 trainable parameters and 384 non-trainable parameters linked to batch normalization layers, as shown in Table 15. This model occupies 1.32 MB of storage and requires approximately 13.1 million floating-point operations (0.013 GFLOPs) for a single forward pass.

**Table 15:** Computational efficiency metrics of the proposed CNN-MHSA model.

Metric	Value
Total parameters	326,273
Trainable parameters	325,889
Non-trainable parameters	384
Model size (.keras)	1.32 MB
FLOPs per single inference	13.1 × 10^6^ (0.013 GFLOPs)
Mean inference latency	13.20 ms
P95 latency	14.73 ms
P99 latency	17.07 ms
Throughput (batch size = 256)	3,008 samples/s
Mean batch processing time	85.12 ms
Peak process memory	894 MB

The average single-sample inference latency was 13.20 ms, and the latency for P95 and P99 was 14.73 ms and 17.07 ms, respectively. The framework was able to process 3,008 samples per second at a batch size of 256, which is equivalent to a mean processing time of 85.12 ms per batch. The model parameter memory requirement was 1.32 MB, and the peak process memory consumption during the inference was about 894 MB, which is mainly caused by the memory requirement of the TensorFlow runtime environment rather than the model parameters themselves.

The findings indicated that the suggested CNN-MHSA architecture has a low computational complexity and is suitable for efficient CPU-based inference in resource-constrained environments. While the model wasn’t deployed on dedicated embedded hardware, the latency, model size, and computational complexity observed indicate that deployment of gateway-based IoMT environments is a viable path to future implementation.

### 4.8 Cross-dataset generalization analysis

To investigate the generalizability of the suggested method beyond a single benchmark, the CNN-MHSA framework was further assessed utilizing the MQTTEEB-D dataset, a real-world MQTT cybersecurity benchmark (Aqachtoul et al., 2025). Unlike CICIoMT2024, which characterizes network traffic using 45 engineered statistical flow features, MQTTEEB-D provides 11 raw and packet-level MQTT/TCP attributes (e.g., tcp_flags, mqtt_hdrflags, and mqtt_qos). Owing to the differences in feature representation between the two datasets, a separate instance of the CNN-MHSA architecture with identical structural configuration was trained and evaluated independently using a stratified 80:20 train-test split. The model was not directly transferred from the CICIoMT2024 dataset because the feature spaces are heterogeneous.

As summarized in Table 16, the proposed architecture attained an accuracy of 91.76%, a precision of 94.56%, a recall of 96.14%, an F1-score of 95.34%, and an AUC of 0.9169 on MQTTEEB-D. Although these results are lower than those obtained on CICIoMT2024, they demonstrate that the proposed architecture maintains strong discriminative capability under substantial distribution shift and differing feature representations.

**Table 16:** Cross-dataset generalization results.

Dataset	Accuracy (%)	Precision (%)	Recall (%)	F1-score (%)	AUC
CICIoMT2024 (MQTT subset)	99.40	99.47	99.58	99.52	0.9998
MQTTEEB-D	91.76	94.56	96.14	95.34	0.9169

The observed performance reduction is expected because MQTTEEB-D contains fewer features and exhibits a substantially different class distribution. The results suggest that the presented CNN-MHSA framework has the potential for transferability across various heterogeneous MQTT intrusion detection datasets.

## 5 Discussion

The experimental findings indicate that the proposed CNN-MHSA framework benefits from the complementary integration of one-dimensional convolution and multi-head self-attention rather than either mechanism operating independently. The convolutional layers first learn hierarchical local representations from statistical network-flow features, while the self-attention module captures long-range contextual dependencies among these learned representations. This integration is especially suitable for MQTT intrusion detection, as malicious activities arise from complex interactions of multiple traffic components rather than individual changes in those components. The ablation study further corroborates this finding, as both the CNN-only and the MHSA-only configurations result in worse performance than the integrated configuration, showing that local feature extraction and global dependency modeling are complementary for protocol-specific intrusion detection.

The proposed framework is also conceptually different from recent transformer-based, recurrent attention-based, and graph neural network models for intrusion detection. The proposed CNN-MHSA framework sequentially combines the lightweight convolutional feature extraction and the global attention modeling, while these architectures focus mainly on the global attention, the temporal sequence learning, or the feature relationships among the graphs. This design is appropriate for the statistical flow representation of CICIoMT2024 and has low computational complexity while effectively representing the features of the statistical flow.

The broader experiments also support the feasibility of the suggested framework. The computational efficiency analysis indicates that the model has a compact architecture that has a low computational overhead, which implies that the inference can be efficiently done on a CPU in the gateway-oriented IoMT environment. The cross-dataset evaluation results also show that the learned feature representations remain effective even when there are significant differences in feature representation and traffic distribution between CICIoMT2024 and MQTTEEB-D, which does not imply the generalizability of the learned feature representations but rather indicates that the proposed architecture offers a robust and transferable framework for protocol-specific MQTT intrusion detection along with dataset-specific training.

## 6 Conclusion

In this work, a CNN-MHSA-based intrusion detection system for the MQTT protocol is proposed to protect IoMT networks and is evaluated on the CICIoMT2024 benchmark dataset using a binary classification approach. To learn local network traffic characteristics and global feature interactions, a hybrid deep learning model based on 1D-CNN and multi-head self-attention is proposed for network flow modeling. The outcome of the experiments shows the effectiveness of the proposed CNN–MHSA architecture for detecting protocol-specific attacks in the context of IoMT networks. The integration of convolutional feature extraction and attention-based representation learning allows the system to discern subtle statistical variations and rich feature connections, crucial in detecting intricate attack patterns. The findings further demonstrate the potential of combining convolutional feature extraction with multi-head self-attention to jointly learn local traffic characteristics and long-range feature dependencies for protocol-specific MQTT intrusion detection under the adopted benchmark setting.

Although the results have been promising, certain challenges remain. Firstly, the present research adopts a binary classification approach and thus cannot cover the wider range of attack categories present in IoMT environments. Second, computational efficiency metrics were quantified using CPU-based inference profiling, but the proposed framework wasn’t tested on dedicated embedded or edge computing platforms. However, the present study does not include factors such as device heterogeneity, energy usage, network differences, and deployment-specific restrictions in real-world applications. Additionally, the efficiency analysis in Section 4.7 was performed in an architectural profiling manner instead of being implemented on physical edge devices. Validation on resource-limited components, such as gateways of the Raspberry Pi class or clinical IoMT devices, remains a major concern for future studies. Furthermore, the proposed framework has not yet been validated using real-world clinical IoMT deployments or operational hospital network environments, and, therefore, its practical performance under real clinical conditions remains to be established. The proposed framework was also tested with the MQTTEEB-D dataset to demonstrate the cross-dataset generalization capability, but further testing on a set of diverse IoMT and MQTT testbeds is required to assess the robustness of the suggested system under diverse traffic conditions and deployment scenarios. In addition, the present study primarily considers large-scale benchmark datasets, and the behavior of the proposed architecture on relatively small or data-limited IoMT datasets remains to be systematically investigated, particularly in scenarios where limited training data may influence deep learning model generalization.

In the future, the proposed system will be expanded to multi-class intrusion detection, which will enable the precise identification of various categories of MQTT attacks. In a multi-class setting, the current CNN-MHSA can be modified by swapping the sigmoid output layer with a softmax output layer and optimizing the model by employing the categorical cross-entropy loss function. Such an extension would allow for attack-specific response mechanisms and make intrusion detection systems more useful in a heterogeneous IoMT environment. Moreover, future research will focus on deployment on embedded IoMT platforms using model compression and optimization methods such as pruning, quantization, TensorFlow Lite conversion, and hardware-aware model architecture refinement.

## Data Availability

The original contributions presented in the study are included in the article/supplementary material, further inquiries can be directed to the corresponding author.
